# 2-[(1*R*,3*S*)-6,7-Dimeth­oxy-1-phenyl-1,2,3,4-tetra­hydro­isoquinolin-3-yl]-4-phenyl-1,3-thia­zole

**DOI:** 10.1107/S1600536811037494

**Published:** 2011-09-30

**Authors:** Sunayna Pawar, Venugopala Katharigatta, Thavendran Govender, Hendrik G. Kruger, Glenn E. M. Maguire

**Affiliations:** a School of Pharmacy and Pharmacology, University of KwaZulu-Natal, Durban 4000, South Africa; b School of Chemistry, University of KwaZulu-Natal, Durban 4000, South Africa

## Abstract

In the title compound, C_26_H_24_N_2_O_2_S, the dihedral angle between the thia­zole ring and the adjacent phenyl ring is 3.02 (15)°. The N-containing six-membered ring of the tetra­hydro­isoquinoline unit adopts a half-chair conformation. The dihedral angle between the least-squares plane of the tetra­hydro­isoquinoline ring system and its nearest phenyl ring is 76.90 (13)°. No classical hydrogen bonds nor π–π inter­actions were found in the crystal structure.

## Related literature

For reactions associated with TIQ ligands, see: Chakka *et al.* (2010 ▶); Naicker *et al.* (2010 ▶). For related structures, see: Naicker *et al.* (2011*a* ▶,*b* ▶).
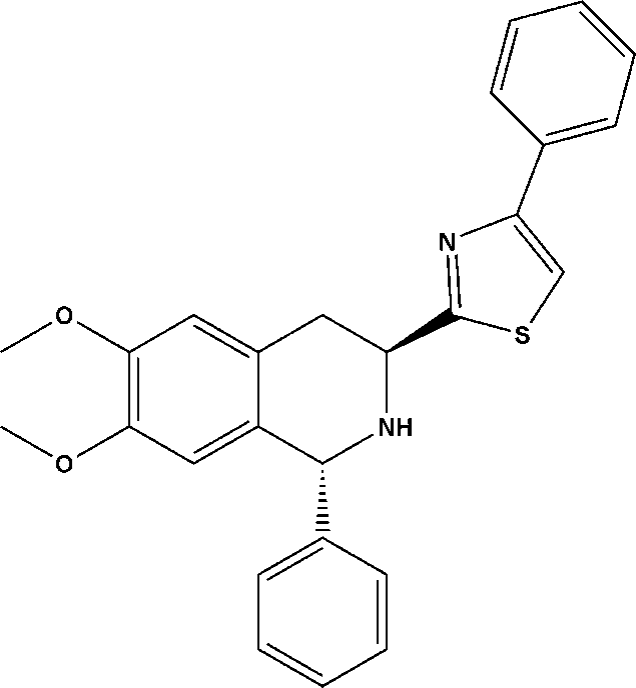


## Experimental

### 

#### Crystal data

C_26_H_24_N_2_O_2_S
*M* *_r_* = 428.53
Orthorhombic, 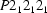

*a* = 5.9178 (2) Å
*b* = 16.6269 (9) Å
*c* = 22.8564 (11) Å
*V* = 2248.95 (18) Å^3^
*Z* = 4
Mo *K*α radiation
μ = 0.17 mm^−1^
*T* = 293 K
0.32 × 0.16 × 0.13 mm

#### Data collection

Nonius KappaCCD diffractometer
Absorption correction: multi-scan (*SADABS*; Sheldrick, 1996 ▶) *T*_min_ = 0.886, *T*_max_ = 0.978
92668 measured reflections
4948 independent reflections
3141 reflections with *I* > 2σ(*I*)
*R*_int_ = 0.093

#### Refinement

*R*[*F* ^2^ > 2σ(*F* ^2^)] = 0.075
*wR*(*F* ^2^) = 0.109
*S* = 1.21
4948 reflections
285 parameters
1 restraint
H atoms treated by a mixture of independent and constrained refinement
Δρ_max_ = 0.18 e Å^−3^
Δρ_min_ = −0.13 e Å^−3^
Absolute structure: Flack (1983 ▶), 2094 Friedel pairs
Flack parameter: 0.01 (10)

### 

Data collection: *COLLECT* (Nonius, 2000 ▶); cell refinement: *DENZO-SMN* (Otwinowski & Minor, 1997 ▶); data reduction: *DENZO-SMN*; program(s) used to solve structure: *SHELXS97* (Sheldrick, 2008 ▶); program(s) used to refine structure: *SHELXL97* (Sheldrick, 2008 ▶); molecular graphics: *OLEX2* (Dolomanov *et al.*, 2009 ▶); software used to prepare material for publication: *SHELXL97*.

## Supplementary Material

Crystal structure: contains datablock(s) I, global. DOI: 10.1107/S1600536811037494/is2779sup1.cif

Structure factors: contains datablock(s) I. DOI: 10.1107/S1600536811037494/is2779Isup2.hkl

Supplementary material file. DOI: 10.1107/S1600536811037494/is2779Isup3.cml

Additional supplementary materials:  crystallographic information; 3D view; checkCIF report

## Figures and Tables

**Fig. 1.**
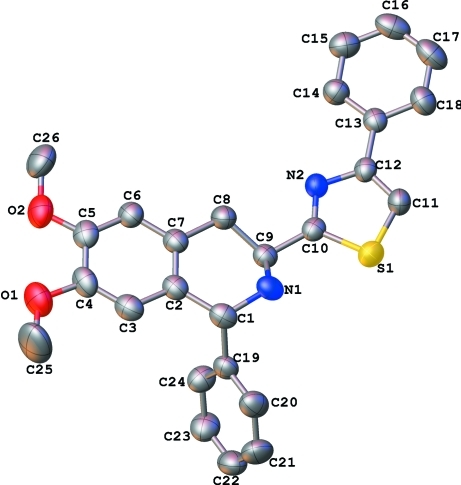
The molecular structure of the title compound with the atom numbering scheme. Displacement ellipsoids are drawn at the 40% probability level. Hydrogen atoms have been omitted for clarity.

## supplementary crystallographic information

### Comment

As a part of our studies on the synthesis and application of new tetrahydroisoquinoline compounds for catalysis of asymmetric hydogenation reactions (Chakka *et al.*, 2010) and the Diels-Alder reaction (Naicker *et al.*, 2010), the title compound, a novel ligand containing a TIQ backbone with thiazole moiety, and its' analogues are currently being tested in our laboratory for the asymmetric Henry reaction.

The absolute stereochemistry was confirmed to be *R* and *S* at C1 and C9 positions, respectively, by two-dimensional NMR studies. From the crystal structure it is evident that the *N*-containing six-membered ring assumes a half chair conformation [Q = 0.446 (4) Å, θ = 54.0 (5)° and φ = 315.6 (6)°]. The torsion angle for C1—N1—C9—C10 is 57.9 (4)°. The maximum displacements from the C1/C2/C7–C9/N1 plane are 0.256 Å for N1 and 0.314 Å for C9 (Fig. 1). This is similar to our previously reported structures which also assume half chair conformations (Naicker *et al.*, 2011*a*, *b*). There are no hydrogen bonding interactions nor π–π interactions in the crystal structure.

### Experimental

A solution of Cbz protected thiazole compound (0.100 g, 0.177 mmol, 1 eq.) in dry DCM (4 ml) under argon atmosphere was treated with dipropylsulfide (0.788 ml, 5.338 mmol, 30 eq.), boron trifluoride diethyl etherate (0.230 ml, 10 eq.) and stirred at room temperature for 1.5 h, then dipropylsulfide (0.499 ml, 2.336 mmol, 20 eq.) was added, the reaction was allowed to proceed for another 2 h. The reaction was monitored by TLC using EtOAc/Hexane (20:80, *R*_f_ = 1/2). The mixture was then poured into water (5 ml) and 10% aqueous NH_4_OH (10 ml) was added and extracted with ethylacetate (30 ml) followed by washing with water (2 × 10 ml). The organic layer was separated and dried over anhydrous MgSO_4_ and the solvent evaporated under reduced pressure to afford crude thiazole, which was purified by column chromatography using silica gel (deactivated with 5% Et_3_N) using 95:5 hexane/Et_3_N - 10:90:5% EtOAc /hexane/Et_3_N as the eluent to yield approximately 0.05 g (65%) of pure thiazole compound. *M*.p. = 388–390 K

Crystals suitable for X-ray analysis were grown at room temperature from a solution of EtOAc/hexane.

### Refinement

All H atoms, except atom H1N, were placed in idealized positions (C—H = 0.93–0.97 Å) and refined using a riding model, with *U*_iso_(H) set at 1.2 or 1.5 times those of their parent atoms. The H1N was refined freely [N1—H1N = 0.927 (17) Å].

### Crystal data

**Table:**

C_26_H_24_N_2_O_2_S	*F*(000) = 904
*M**_r_* = 428.53	*D*_x_ = 1.266 Mg m^−^^3^
Orthorhombic, *P*2_1_2_1_2_1_	Mo *K*α radiation, λ = 0.71073 Å
Hall symbol: P 2ac 2ab	Cell parameters from 92668 reflections
*a* = 5.9178 (2) Å	θ = 3.0–27.1°
*b* = 16.6269 (9) Å	µ = 0.17 mm^−^^1^
*c* = 22.8564 (11) Å	*T* = 293 K
*V* = 2248.95 (18) Å^3^	Block, colourless
*Z* = 4	0.32 × 0.16 × 0.13 mm

### Data collection

**Table:**

Nonius KappaCCD diffractometer	4948 independent reflections
Radiation source: fine-focus sealed tube	3141 reflections with *I* > 2σ(*I*)
graphite	*R*_int_ = 0.093
1.2° φ scans and ω scans	θ_max_ = 27.1°, θ_min_ = 3.0°
Absorption correction: multi-scan (*SADABS*; Sheldrick, 1996)	*h* = −7→7
*T*_min_ = 0.886, *T*_max_ = 0.978	*k* = −21→21
92668 measured reflections	*l* = −29→29

### Refinement

**Table:**

Refinement on *F*^2^	Hydrogen site location: inferred from neighbouring sites
Least-squares matrix: full	H atoms treated by a mixture of independent and constrained refinement
*R*[*F*^2^ > 2σ(*F*^2^)] = 0.075	*w* = 1/[σ^2^(*F*_o_^2^) + (0.0259*P*)^2^ + 0.4897*P*] where *P* = (*F*_o_^2^ + 2*F*_c_^2^)/3
*wR*(*F*^2^) = 0.109	(Δ/σ)_max_ = 0.001
*S* = 1.21	Δρ_max_ = 0.18 e Å^−^^3^
4948 reflections	Δρ_min_ = −0.13 e Å^−^^3^
285 parameters	Extinction correction: *SHELXL97* (Sheldrick, 2008), Fc^*^=kFc[1+0.001xFc^2^λ^3^/sin(2θ)]^-1/4^
1 restraint	Extinction coefficient: 0.0072 (9)
Primary atom site location: structure-invariant direct methods	Absolute structure: Flack (1983), 2094 Friedel pairs
Secondary atom site location: difference Fourier map	Flack parameter: 0.01 (10)

### Special details

**Table:**

Geometry. All e.s.d.'s (except the e.s.d. in the dihedral angle between two l.s. planes) are estimated using the full covariance matrix. The cell e.s.d.'s are taken into account individually in the estimation of e.s.d.'s in distances, angles and torsion angles; correlations between e.s.d.'s in cell parameters are only used when they are defined by crystal symmetry. An approximate (isotropic) treatment of cell e.s.d.'s is used for estimating e.s.d.'s involving l.s. planes.
Refinement. Refinement of *F*^2^ against ALL reflections. The weighted *R*-factor *wR* and goodness of fit *S* are based on *F*^2^, conventional *R*-factors *R* are based on *F*, with *F* set to zero for negative *F*^2^. The threshold expression of *F*^2^ > σ(*F*^2^) is used only for calculating *R*-factors(gt) *etc*. and is not relevant to the choice of reflections for refinement. *R*-factors based on *F*^2^ are statistically about twice as large as those based on *F*, and *R*- factors based on ALL data will be even larger.

### Fractional atomic coordinates and isotropic or equivalent isotropic displacement parameters (Å^2^)

**Table:**

	*x*	*y*	*z*	*U*_iso_*/*U*_eq_	
S1	0.31297 (13)	0.27081 (5)	0.16545 (4)	0.0756 (3)	
O1	1.3723 (4)	0.11255 (15)	0.43058 (11)	0.0981 (8)	
O2	1.4727 (4)	0.02633 (14)	0.34014 (11)	0.0864 (7)	
N1	0.5823 (4)	0.23535 (15)	0.27440 (10)	0.0623 (6)	
H1N	0.490 (4)	0.1910 (13)	0.2803 (11)	0.069 (9)*	
N2	0.6162 (4)	0.18000 (14)	0.12153 (10)	0.0564 (6)	
C1	0.7220 (5)	0.23931 (17)	0.32849 (12)	0.0620 (7)	
H1	0.6242	0.2210	0.3604	0.074*	
C2	0.9256 (5)	0.18372 (17)	0.32871 (13)	0.0595 (7)	
C3	1.0545 (6)	0.17525 (19)	0.37999 (13)	0.0708 (9)	
H3	1.0166	0.2054	0.4129	0.085*	
C4	1.2354 (6)	0.1235 (2)	0.38266 (14)	0.0717 (9)	
C5	1.2916 (5)	0.07670 (17)	0.33380 (15)	0.0663 (8)	
C6	1.1660 (5)	0.08514 (16)	0.28383 (14)	0.0643 (8)	
H6	1.2022	0.0542	0.2512	0.077*	
C7	0.9852 (5)	0.13892 (16)	0.28054 (12)	0.0590 (7)	
C8	0.8571 (5)	0.14618 (16)	0.22391 (13)	0.0651 (8)	
H8A	0.7586	0.1000	0.2195	0.078*	
H8B	0.9632	0.1461	0.1916	0.078*	
C9	0.7172 (4)	0.22227 (16)	0.22165 (11)	0.0552 (7)	
H9	0.8209	0.2679	0.2175	0.066*	
C10	0.5672 (4)	0.22039 (16)	0.16855 (12)	0.0542 (7)	
C11	0.2745 (5)	0.23434 (18)	0.09645 (12)	0.0668 (8)	
H11	0.1493	0.2455	0.0732	0.080*	
C12	0.4483 (5)	0.18743 (16)	0.07989 (12)	0.0543 (7)	
C13	0.4750 (5)	0.14401 (16)	0.02377 (12)	0.0556 (7)	
C14	0.6676 (6)	0.09961 (18)	0.01269 (14)	0.0673 (8)	
H14	0.7819	0.0980	0.0406	0.081*	
C15	0.6937 (7)	0.0574 (2)	−0.03919 (15)	0.0804 (10)	
H15	0.8243	0.0277	−0.0460	0.096*	
C16	0.5244 (8)	0.0598 (2)	−0.08076 (16)	0.0882 (11)	
H16	0.5400	0.0315	−0.1156	0.106*	
C17	0.3339 (7)	0.1041 (2)	−0.07042 (17)	0.0935 (11)	
H17	0.2209	0.1064	−0.0987	0.112*	
C18	0.3074 (6)	0.1455 (2)	−0.01871 (14)	0.0746 (9)	
H18	0.1758	0.1748	−0.0122	0.089*	
C19	0.7776 (5)	0.32691 (17)	0.34164 (12)	0.0592 (7)	
C20	0.6135 (6)	0.3740 (2)	0.36789 (14)	0.0801 (10)	
H20	0.4733	0.3518	0.3767	0.096*	
C21	0.6556 (7)	0.4538 (2)	0.38112 (16)	0.0941 (12)	
H21	0.5430	0.4849	0.3983	0.113*	
C22	0.8609 (7)	0.4872 (2)	0.36917 (15)	0.0852 (11)	
H22	0.8902	0.5405	0.3791	0.102*	
C23	1.0220 (6)	0.44213 (19)	0.34258 (16)	0.0833 (10)	
H23	1.1607	0.4652	0.3333	0.100*	
C24	0.9825 (5)	0.36207 (18)	0.32905 (13)	0.0707 (8)	
H24	1.0956	0.3318	0.3113	0.085*	
C25	1.3075 (9)	0.1475 (3)	0.48294 (17)	0.153 (2)	
H25A	1.4160	0.1346	0.5127	0.230*	
H25B	1.1618	0.1274	0.4942	0.230*	
H25C	1.2998	0.2048	0.4782	0.230*	
C26	1.5162 (7)	−0.0274 (2)	0.29331 (17)	0.1042 (12)	
H26A	1.6461	−0.0596	0.3024	0.156*	
H26B	1.5445	0.0028	0.2582	0.156*	
H26C	1.3876	−0.0616	0.2875	0.156*	

### Atomic displacement parameters (Å^2^)

**Table:**

	*U*^11^	*U*^22^	*U*^33^	*U*^12^	*U*^13^	*U*^23^
S1	0.0669 (5)	0.0765 (5)	0.0833 (6)	0.0138 (4)	−0.0023 (5)	−0.0113 (5)
O1	0.108 (2)	0.1159 (19)	0.0704 (16)	−0.0031 (16)	−0.0193 (15)	0.0182 (14)
O2	0.0864 (16)	0.0804 (15)	0.0922 (17)	0.0051 (14)	−0.0139 (14)	0.0272 (14)
N1	0.0589 (14)	0.0688 (16)	0.0591 (15)	−0.0141 (13)	0.0045 (13)	−0.0059 (13)
N2	0.0537 (14)	0.0571 (14)	0.0586 (15)	−0.0013 (12)	−0.0042 (12)	0.0025 (12)
C1	0.0660 (17)	0.0680 (18)	0.0521 (17)	−0.0134 (16)	0.0078 (15)	0.0001 (15)
C2	0.0680 (18)	0.0550 (16)	0.0553 (19)	−0.0132 (15)	−0.0013 (16)	0.0076 (15)
C3	0.088 (2)	0.067 (2)	0.0572 (19)	−0.015 (2)	−0.0008 (19)	0.0066 (16)
C4	0.083 (2)	0.075 (2)	0.058 (2)	−0.0156 (19)	−0.0130 (18)	0.0236 (18)
C5	0.072 (2)	0.0569 (18)	0.070 (2)	−0.0090 (17)	−0.004 (2)	0.0211 (17)
C6	0.073 (2)	0.0554 (17)	0.065 (2)	−0.0027 (17)	−0.0031 (18)	0.0074 (15)
C7	0.0682 (19)	0.0512 (16)	0.0577 (18)	−0.0083 (16)	−0.0037 (16)	0.0059 (15)
C8	0.073 (2)	0.0579 (17)	0.0642 (18)	0.0008 (16)	−0.0060 (16)	−0.0030 (15)
C9	0.0561 (16)	0.0525 (16)	0.0569 (17)	−0.0083 (14)	0.0004 (14)	0.0011 (14)
C10	0.0524 (15)	0.0470 (15)	0.0632 (18)	−0.0039 (13)	0.0024 (15)	0.0046 (16)
C11	0.0632 (19)	0.0667 (18)	0.071 (2)	0.0064 (17)	−0.0118 (16)	0.0021 (16)
C12	0.0542 (17)	0.0480 (16)	0.0608 (19)	−0.0026 (14)	−0.0030 (15)	0.0087 (14)
C13	0.0574 (18)	0.0494 (16)	0.0601 (18)	−0.0087 (15)	−0.0013 (15)	0.0080 (14)
C14	0.070 (2)	0.0705 (19)	0.062 (2)	−0.0012 (18)	0.0015 (17)	−0.0013 (16)
C15	0.085 (2)	0.071 (2)	0.085 (3)	−0.004 (2)	0.011 (2)	−0.0061 (19)
C16	0.100 (3)	0.089 (3)	0.076 (3)	−0.035 (2)	0.007 (2)	−0.019 (2)
C17	0.091 (3)	0.113 (3)	0.077 (3)	−0.029 (3)	−0.017 (2)	−0.011 (2)
C18	0.0670 (19)	0.084 (2)	0.073 (2)	−0.0108 (19)	−0.0110 (19)	0.0016 (18)
C19	0.0670 (19)	0.0643 (18)	0.0465 (16)	−0.0024 (16)	−0.0002 (15)	−0.0030 (14)
C20	0.070 (2)	0.091 (3)	0.079 (2)	−0.002 (2)	0.0061 (17)	−0.017 (2)
C21	0.102 (3)	0.085 (3)	0.095 (3)	0.016 (2)	0.004 (2)	−0.029 (2)
C22	0.108 (3)	0.063 (2)	0.084 (3)	−0.002 (2)	−0.015 (2)	−0.0087 (18)
C23	0.082 (2)	0.065 (2)	0.103 (3)	−0.0104 (19)	−0.002 (2)	0.008 (2)
C24	0.069 (2)	0.0606 (19)	0.082 (2)	−0.0033 (17)	0.0095 (18)	0.0037 (17)
C25	0.166 (4)	0.223 (6)	0.071 (3)	0.033 (5)	−0.032 (3)	−0.015 (3)
C26	0.108 (3)	0.080 (2)	0.125 (3)	0.022 (2)	−0.005 (3)	0.017 (2)

### Geometric parameters (Å, °)

**Table:**

S1—C11	1.705 (3)	C12—C13	1.480 (4)
S1—C10	1.724 (3)	C13—C14	1.381 (4)
O1—C4	1.374 (4)	C13—C18	1.388 (4)
O1—C25	1.385 (4)	C14—C15	1.386 (4)
O2—C5	1.368 (4)	C14—H14	0.9300
O2—C26	1.417 (4)	C15—C16	1.381 (5)
N1—C9	1.462 (3)	C15—H15	0.9300
N1—C1	1.489 (3)	C16—C17	1.368 (5)
N1—H1N	0.927 (17)	C16—H16	0.9300
N2—C10	1.300 (3)	C17—C18	1.376 (5)
N2—C12	1.381 (3)	C17—H17	0.9300
C1—C2	1.519 (4)	C18—H18	0.9300
C1—C19	1.523 (4)	C19—C24	1.377 (4)
C1—H1	0.9800	C19—C20	1.385 (4)
C2—C7	1.375 (4)	C20—C21	1.382 (5)
C2—C3	1.406 (4)	C20—H20	0.9300
C3—C4	1.376 (4)	C21—C22	1.364 (5)
C3—H3	0.9300	C21—H21	0.9300
C4—C5	1.401 (4)	C22—C23	1.357 (5)
C5—C6	1.370 (4)	C22—H22	0.9300
C6—C7	1.397 (4)	C23—C24	1.386 (4)
C6—H6	0.9300	C23—H23	0.9300
C7—C8	1.505 (4)	C24—H24	0.9300
C8—C9	1.513 (4)	C25—H25A	0.9600
C8—H8A	0.9700	C25—H25B	0.9600
C8—H8B	0.9700	C25—H25C	0.9600
C9—C10	1.504 (4)	C26—H26A	0.9600
C9—H9	0.9800	C26—H26B	0.9600
C11—C12	1.345 (4)	C26—H26C	0.9600
C11—H11	0.9300		
			
C11—S1—C10	88.95 (14)	C11—C12—C13	127.4 (3)
C4—O1—C25	118.1 (3)	N2—C12—C13	118.5 (2)
C5—O2—C26	116.6 (3)	C14—C13—C18	118.1 (3)
C9—N1—C1	112.8 (2)	C14—C13—C12	120.5 (3)
C9—N1—H1N	108.9 (17)	C18—C13—C12	121.4 (3)
C1—N1—H1N	103.9 (17)	C13—C14—C15	121.3 (3)
C10—N2—C12	111.3 (2)	C13—C14—H14	119.4
N1—C1—C2	114.6 (2)	C15—C14—H14	119.4
N1—C1—C19	109.0 (2)	C16—C15—C14	119.5 (4)
C2—C1—C19	114.2 (2)	C16—C15—H15	120.2
N1—C1—H1	106.1	C14—C15—H15	120.2
C2—C1—H1	106.1	C17—C16—C15	119.7 (3)
C19—C1—H1	106.1	C17—C16—H16	120.2
C7—C2—C3	118.3 (3)	C15—C16—H16	120.2
C7—C2—C1	122.0 (3)	C16—C17—C18	120.8 (4)
C3—C2—C1	119.6 (3)	C16—C17—H17	119.6
C4—C3—C2	121.5 (3)	C18—C17—H17	119.6
C4—C3—H3	119.3	C17—C18—C13	120.7 (3)
C2—C3—H3	119.3	C17—C18—H18	119.7
O1—C4—C3	125.2 (3)	C13—C18—H18	119.7
O1—C4—C5	115.0 (3)	C24—C19—C20	117.9 (3)
C3—C4—C5	119.8 (3)	C24—C19—C1	123.7 (3)
O2—C5—C6	125.2 (3)	C20—C19—C1	118.4 (3)
O2—C5—C4	116.2 (3)	C21—C20—C19	120.7 (3)
C6—C5—C4	118.6 (3)	C21—C20—H20	119.6
C5—C6—C7	121.7 (3)	C19—C20—H20	119.6
C5—C6—H6	119.1	C22—C21—C20	120.5 (4)
C7—C6—H6	119.1	C22—C21—H21	119.7
C2—C7—C6	120.0 (3)	C20—C21—H21	119.7
C2—C7—C8	121.1 (3)	C23—C22—C21	119.3 (3)
C6—C7—C8	118.9 (3)	C23—C22—H22	120.3
C7—C8—C9	111.8 (2)	C21—C22—H22	120.3
C7—C8—H8A	109.2	C22—C23—C24	120.8 (3)
C9—C8—H8A	109.2	C22—C23—H23	119.6
C7—C8—H8B	109.2	C24—C23—H23	119.6
C9—C8—H8B	109.2	C19—C24—C23	120.6 (3)
H8A—C8—H8B	107.9	C19—C24—H24	119.7
N1—C9—C10	110.3 (2)	C23—C24—H24	119.7
N1—C9—C8	113.3 (2)	O1—C25—H25A	109.5
C10—C9—C8	109.5 (2)	O1—C25—H25B	109.5
N1—C9—H9	107.9	H25A—C25—H25B	109.5
C10—C9—H9	107.9	O1—C25—H25C	109.5
C8—C9—H9	107.9	H25A—C25—H25C	109.5
N2—C10—C9	123.1 (2)	H25B—C25—H25C	109.5
N2—C10—S1	114.3 (2)	O2—C26—H26A	109.5
C9—C10—S1	122.6 (2)	O2—C26—H26B	109.5
C12—C11—S1	111.4 (2)	H26A—C26—H26B	109.5
C12—C11—H11	124.3	O2—C26—H26C	109.5
S1—C11—H11	124.3	H26A—C26—H26C	109.5
C11—C12—N2	114.1 (2)	H26B—C26—H26C	109.5
			
C9—N1—C1—C2	−35.5 (3)	C8—C9—C10—N2	−26.3 (3)
C9—N1—C1—C19	94.0 (3)	N1—C9—C10—S1	27.5 (3)
N1—C1—C2—C7	5.4 (3)	C8—C9—C10—S1	152.8 (2)
C19—C1—C2—C7	−121.4 (3)	C11—S1—C10—N2	0.0 (2)
N1—C1—C2—C3	−172.5 (2)	C11—S1—C10—C9	−179.2 (2)
C19—C1—C2—C3	60.6 (3)	C10—S1—C11—C12	0.3 (2)
C7—C2—C3—C4	−0.3 (4)	S1—C11—C12—N2	−0.4 (3)
C1—C2—C3—C4	177.7 (2)	S1—C11—C12—C13	178.9 (2)
C25—O1—C4—C3	9.3 (5)	C10—N2—C12—C11	0.4 (3)
C25—O1—C4—C5	−170.6 (4)	C10—N2—C12—C13	−179.0 (2)
C2—C3—C4—O1	178.9 (3)	C11—C12—C13—C14	178.3 (3)
C2—C3—C4—C5	−1.2 (4)	N2—C12—C13—C14	−2.4 (4)
C26—O2—C5—C6	−7.4 (4)	C11—C12—C13—C18	−2.7 (4)
C26—O2—C5—C4	173.5 (3)	N2—C12—C13—C18	176.6 (3)
O1—C4—C5—O2	0.4 (4)	C18—C13—C14—C15	−0.2 (4)
C3—C4—C5—O2	−179.6 (2)	C12—C13—C14—C15	178.9 (3)
O1—C4—C5—C6	−178.8 (2)	C13—C14—C15—C16	0.1 (5)
C3—C4—C5—C6	1.3 (4)	C14—C15—C16—C17	0.4 (5)
O2—C5—C6—C7	−179.0 (2)	C15—C16—C17—C18	−0.9 (6)
C4—C5—C6—C7	0.1 (4)	C16—C17—C18—C13	0.9 (5)
C3—C2—C7—C6	1.7 (4)	C14—C13—C18—C17	−0.3 (5)
C1—C2—C7—C6	−176.3 (2)	C12—C13—C18—C17	−179.3 (3)
C3—C2—C7—C8	−178.9 (2)	N1—C1—C19—C24	−101.3 (3)
C1—C2—C7—C8	3.2 (4)	C2—C1—C19—C24	28.4 (4)
C5—C6—C7—C2	−1.6 (4)	N1—C1—C19—C20	78.9 (3)
C5—C6—C7—C8	178.9 (3)	C2—C1—C19—C20	−151.4 (3)
C2—C7—C8—C9	17.6 (4)	C24—C19—C20—C21	−0.2 (5)
C6—C7—C8—C9	−162.9 (2)	C1—C19—C20—C21	179.6 (3)
C1—N1—C9—C10	−179.0 (2)	C19—C20—C21—C22	−0.8 (5)
C1—N1—C9—C8	58.0 (3)	C20—C21—C22—C23	1.9 (6)
C7—C8—C9—N1	−48.1 (3)	C21—C22—C23—C24	−1.9 (5)
C7—C8—C9—C10	−171.6 (2)	C20—C19—C24—C23	0.2 (4)
C12—N2—C10—C9	178.9 (2)	C1—C19—C24—C23	−179.6 (3)
C12—N2—C10—S1	−0.2 (3)	C22—C23—C24—C19	0.9 (5)
N1—C9—C10—N2	−151.6 (2)		

## References

[bb1] Chakka, S. K., Andersson, P. G., Maguire, G. E. M., Kruger, H. G. & Govender, T. (2010). *Eur. J. Org. Chem.* pp. 972–980.

[bb2] Dolomanov, O. V., Bourhis, L. J., Gildea, R. J., Howard, J. A. K. & Puschmann, H. (2009). *J. Appl. Cryst.* **42**, 339–341.

[bb3] Flack, H. D. (1983). *Acta Cryst.* A**39**, 876–881.

[bb4] Naicker, T., Govender, T., Kruger, H. G. & Maguire, G. E. M. (2011*a*). *Acta Cryst.* E**67**, o67. 10.1107/S1600536810050361 PMC3050362 21522778

[bb5] Naicker, T., Govender, T., Kruger, H. G. & Maguire, G. E. M. (2011*b*). *Acta Cryst.* E**67**, o1403. 10.1107/S1600536811017430 PMC3120496 21754788

[bb6] Naicker, T., Petzold, K., Singh, T., Arvidsson, P. I., Kruger, H. G., Maguire, G. E. M. & Govender, T. (2010). *Tetrahedron Asymmetry*, **21**, 2859–2867.

[bb7] Nonius (2000). *COLLECT* Nonius BV, Delft, The Netherlands.

[bb8] Otwinowski, Z. & Minor, W. (1997). *Methods in Enzymology*, Vol. 276, *Macromolecular Crystallography*, Part A, edited by C. W. Carter Jr & R. M. Sweet, pp. 307–326. New York: Academic Press.

[bb9] Sheldrick, G. M. (1996). *SADABS* University of Göttingen, Germany.

[bb10] Sheldrick, G. M. (2008). *Acta Cryst.* A**64**, 112–122. 10.1107/S0108767307043930 18156677

